# Prognostic and Immunological Value of Angiotensin-Converting Enzyme 2 in Pan-Cancer

**DOI:** 10.3389/fmolb.2020.00189

**Published:** 2020-09-01

**Authors:** Huan Feng, Xian Wei, Linhao Pang, Yue Wu, Bintao Hu, Yajun Ruan, Zhuo Liu, Jihong Liu, Tao Wang

**Affiliations:** ^1^Department of Urology, Tongji Hospital, Tongji Medical College, Huazhong University of Science and Technology, Wuhan, China; ^2^Institute of Urology, Tongji Hospital, Tongji Medical College, Huazhong University of Science and Technology, Wuhan, China

**Keywords:** angiotensin-converting enzyme 2, pan-cancer, database, survival analysis, immune infiltration

## Abstract

Angiotensin-converting enzyme 2 (ACE2) plays a pivotal role in the renin–angiotensin system and is closely related to coronavirus disease of 2019. However, the role of ACE2 in cancers remains unclear. We explored the pan-cancer expression patterns and prognostic value of ACE2 across multiple databases, including Oncomine, PrognoScan, Gene Expression Profiling Interactive Analysis, and Kaplan–Meier Plotter. Then, we investigated the correlations between ACE2 expression and immune infiltration in cancers. We found that tumor tissues had higher expression levels of ACE2 compared with normal tissue in the kidney and the liver and lower expression levels in the lung. High expression levels of ACE2 were beneficial to survival in ovarian serous cystadenocarcinoma, liver hepatocellular carcinoma, kidney renal papillary cell carcinoma, and kidney renal clear cell carcinoma, although this was not the case in lung squamous cell carcinoma. For those with a better prognosis, there were significant positive correlations between ACE2 expression and immune infiltrates, including B cells, CD8 + T cells, CD4 + T cells, neutrophils, macrophages, and dendritic cells. In conclusion, ACE2 could serve as a pan-cancer prognostic biomarker and is correlated with immune infiltrates.

## Introduction

Cancer is a major cause of morbidity and mortality worldwide, creating huge health and economic burden on society (Siegel et al., 2019). Unfortunately, this burden is expected to further increase owing to the growth in numbers of newly diagnosed cases. By 2030, 22 million new cancer cases and 13 million cancer-related deaths are expected each year (Ferlay et al., 2015). Despite many treatment options, including surgery, chemotherapy, radiation therapy, targeted therapy, and immunotherapy, cancer-related mortality remains unsatisfactory. Therefore, it is particularly important to seek other therapeutic targets for cancer diagnosis and treatment actively.

Angiotensin-converting enzyme 2 is a homolog of carboxypeptidase ACE and forms an important component of the renin–angiotensin system (RAS) (Burrell et al., 2004). This secreted protein catalyzes the cleavage of angiotensin I into angiotensins 1–9 and angiotensin II into the vasodilator angiotensins 1–7 (Vickers et al., 2002). In addition to RAS, ACE2 can hydrolyze peptides from other systems, including neurotensins 1–13, apelin 13, dynorphins 1–13, and some kinin metabolites (Vickers et al., 2002). ACE2 was initially described as ACEH (or ACEH2) in a human lymphoma complementary DNA library (Tipnis et al., 2000) and as ACE2 in a human HF ventricular complementary DNA library. Expression of the ACE2 gene has been found in the heart, kidney, testis, upper airways, lungs, gut, and liver (Donoghue et al., 2003). Abnormalities of ACE2 are associated with cardiovascular disease, cerebrovascular disease, kidney diseases, and diabetes (Sluimer et al., 2008; Tikellis et al., 2012; Anguiano et al., 2017). Also, ACE2 is a functional receptor for a variety of coronaviruses, including severe acute respiratory syndrome -associated coronavirus (SARS-CoV), human coronavirus NL63, and SARS-CoV-2 (Li et al., 2003; Hofmann et al., 2005; Zhou et al., 2020). This suggests that genes related to these viruses could be associated with tumors, as many viruses cause cancer. It has been reported that overexpression of ACE2 may inhibit invasion and angiogenesis in non-small-cell lung cancer (Feng et al., 2011). However, some studies have shown that ACE2 promotes epithelial–mesenchymal transition in renal tubular cells (Burns et al., 2010). Therefore, the mechanisms underlying these contradictory effects of ACE2 on cancer need further study.

The tumor microenvironment (TME) is a complex structure composed of tumor cells, blood vessels, extracellular matrix, and other non-malignant cells (Hui and Chen, 2015). Among these non-malignant cells, immune cells have an important role in the TME. Increasing evidence indicates that interactions between tumor cells and the host’s immune system promote the immune escape of the tumor, eventually leading to its spread, recurrence, and metastasis. For example, tumor cells can upregulate programmed cell death 1 ligand expression and cause programmed cell death receptor 1-positive T cells to undergo apoptosis, disable, and deplete (Zou et al., 2016). Hepatocellular carcinoma cells can reduce the cytotoxicity of T cells and natural killer cells by secreting exosomes and increasing immunosuppressive cells such as M2 macrophages, N2 neutrophils, and regulatory B cells (Han et al., 2019). However, immunotherapies have not yet achieved breakthrough results in cancer treatment. Therefore, finding new targets is key to improving immunotherapies.

In this study, we analyzed the expression of ACE2 in various tumors using Oncomine and the Tumor Immunity Estimate Resource (TIMER). Then, the expression of ACE2 and its correlation with cancer prognosis were analyzed using PrognoScan, Gene Expression Profiling Interactive Analysis (GEPIA), and Kaplan–Meier Plotter. Moreover, we explored the relationship between ACE2 expression and immune cells and cytokines using the TIMER and GEPIA databases. The findings of this study suggest that ACE2 may affect the prognosis of cancer patients via interactions with infiltrating immune cells.

## Materials and Methods

### Oncomine Database Analysis

Oncomine^1^, a database that collects all published cancer microarray data and performs standard analysis, was used to analyze the expression levels of the ACE2 gene in various tumors (Rhodes et al., 2007). The following threshold values were used: *P*-value 0.001 and fold change 1.5.

### Survival Analysis in PrognoScan, Kaplan–Meier Plotter, and Gene Expression Profiling Interactive Analysis

For more rigorous results, we analyzed the correlations of ACE2 expression with survival in various cancer types using PrognoScan^2^, Kaplan–Meier Plotter^3^, and GEPIA^4^ (Mizuno et al., 2009; Tang et al., 2017; Nagy et al., 2018). PrognoScan searches the relations between gene expression and aspects of patient prognosis, including OS and DFS, across a large collection of publicly available cancer microarray datasets. The threshold was adjusted to Cox *P*-value < 0.05, and the “forestplot” package in the R software (version 3.6.2^5^) was used to summarize and visualize the survival analysis from PrognoScan. Kaplan–Meier Plotter, which includes gene chip and RNA sequencing datasets from the Gene Expression Omnibus, European Genome-phenome Archive, and The Cancer Genome Atlas, was used to assess the effects of 54,000 genes on survival in 21 cancer types. Kaplan–Meier Plotter analyzed the relationship of ACE2 expression with OS and RFS in various cancer types, and hazard ratio (HR) values with 95% confidence intervals and log-rank *P*-values were calculated. GEPIA, a web-based tool containing 9,736 tumor samples and 8,587 normal samples based on TCGA and Genotype Tissue Expression data, provides differential expression analysis, correlation analysis, and patient survival analysis. In the present study, GEPIA was used to analyze the effects of ACE2 expression on OS and DFS.

### Correlation Analysis Between Angiotensin-Converting Enzyme 2 and Immune Infiltrates by Tumor Immunity Estimate Resource and Gene Expression Profiling Interactive Analysis

TIMER^6^ provides a user-friendly web interface for dynamic analysis of immune infiltrates across diverse cancer types (Li et al., 2016, 2017). TIMER calculates levels of six infiltrating tumor immune subgroups from 10,897 tumors of 32 cancer types. We analyzed the expression of ACE2 in different cancer types and determined the correlations between ACE2 expression and a large number of immune infiltrates, including B cells, CD4 + T cells, CD8 + T cells, neutrophils, macrophages, and dendritic cells. Relationships between gene expression and tumor purity were also examined. Tumor purity is the proportion of tumor cells in tumor tissue. Its value depends largely on the method used to obtain the tumor tissue; the purity of standard tumor surgery samples is usually less than 70%. Variation in tumor purity can lead to systematic bias in tumor research results based on genomic analysis, and assessment of tumor purity can reduce analysis bias (Rhee et al., 2018). We also analyzed the correlations between ACE2 expression and different subtypes of immune cell markers, including gene markers of B cells, CD8 + T cells, follicular helper T cells, T-helper 1 (Th1) cells, Th2 cells, Th9 cells, Th17 cells, Th22 cells, regulatory T cells, regulatory B cells, exhausted T cells, M1 macrophages, M2 macrophages, tumor-associated macrophages, monocytes, natural killer cells, neutrophils, and dendritic cells. The gene markers of tumor-infiltrating immune cells were selected from Cell Signaling Technology^7^ and R&D Systems^8^ websites. The gene expression level was displayed as log2 RSEM. The x-axis represents ACE2, and the y-axis represents related marker genes. To further verify the significantly correlated markers identified using TIMER, gene expression correlation analysis was performed again in GEPIA. The Spearman method was used to determine the correlation coefficients. ACE2 was displayed on the x-axis, whereas other genes of interest were displayed on the y-axis.

### Statistical Analysis

ACE2 expression levels were determined using Oncomine and GEPIA. The results from Oncomine are displayed with *P*-values, fold changes, and ranks. The results from GEPIA use asterisks to represent *P*-values. PrognoScan, Kaplan–Meier Plotter, and GEPIA were used to obtain survival curves; the results are displayed with HR and *P*-values or Cox *P*-values from log-rank tests. The correlation of gene expression was evaluated using Spearman’s correlation. A *P*-value of less than 0.05 was considered to indicate a significant difference.

## Results

### Pan-Cancer Messenger RNA Expression Levels of Angiotensin-Converting Enzyme 2

**FIGURE 1:**
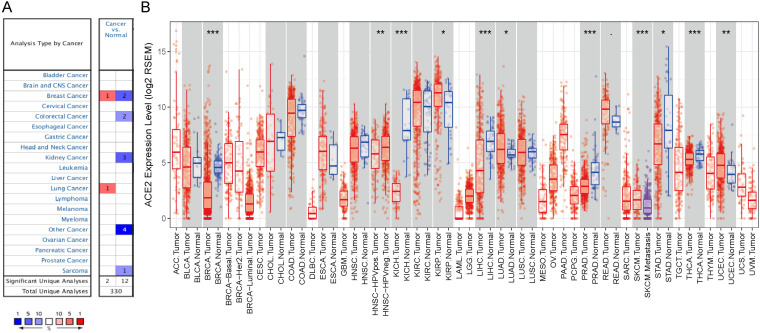
ACE2 expression levels in cancers. **(A)** Increased or decreased expression of ACE2 in different cancer tissues, compared with normal tissues in Oncomine. Number in each cell is the amounts of datasets. **(B)** Human ACE2 expression levels in different cancer types from TCGA data in TIMER (<0.1, *P < 0.05, **P < 0.01, ***P < 0.001).

First, we used the Oncomine database to analyze the messenger RNA expression levels of ACE2 in various cancers. According to the results, compared with normal tissues, the expression of ACE2 was reduced in breast cancer, colorectal cancer, and breast cancer (Figure 1A).

To further examine the pan-cancer expression of ACE2, we explored RNA sequencing data from TCGA in TIMER. Figure 1B shows the differential expression of ACE2 between tumors and adjacent tissues. ACE2 expression levels in BRCA, KICH, LIHC, PRAD, STAD, and thyroid carcinoma were significantly lower than those in adjacent normal tissues. By contrast, ACE2 expression was higher in KIRP, LUAD, and UCEC than in normal tissues.

### Pan-Cancer Prognostic Value of Angiotensin-Converting Enzyme 2

**FIGURE 2:**
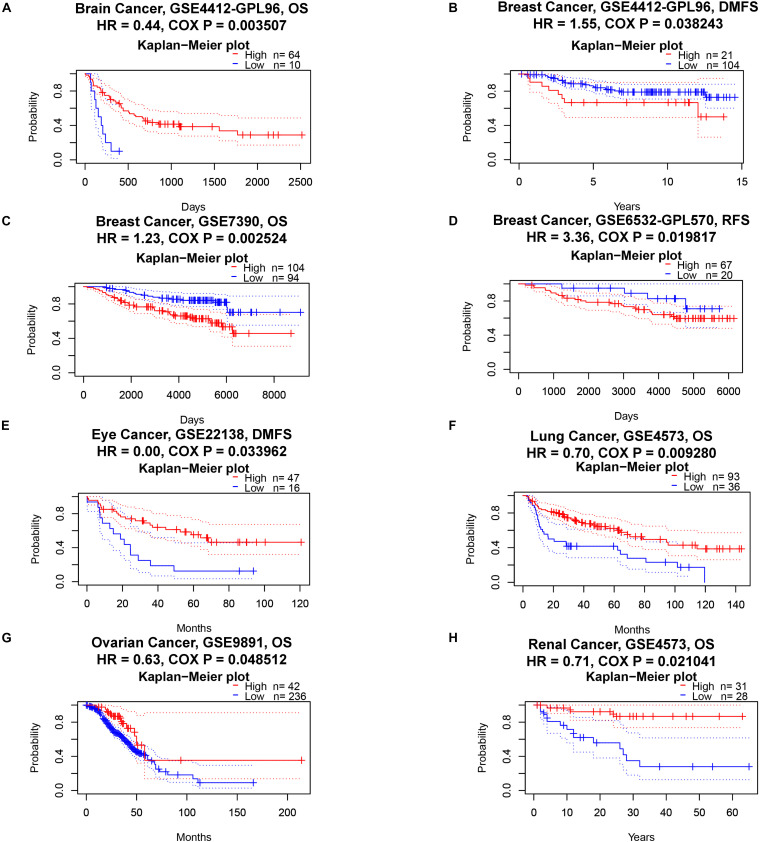
Kaplan–Meier survival curves comparing high and low expression of ACE2 in different cancer types in PrognoScan. OS, overall survival; DMFS, distant metastasis-free survival; RFS, relapse-free survival. **(A)** OS of Brain Cancer. **(B–D)** DMFS, OS and RFS of Breast Cancer. **(E)** DMFS of Eye Cancer. **(F)** OS of Lung Cancer. **(G)** OS of Ovarian Cancer. **(H)** OS of Renal Cancer.

Next, we analyzed the pan-cancer prognostic value of ACE2 using different databases. In PrognoScan, we explored the relationship between the expression of ACE2 and the prognosis of various cancers; the results are shown in Supplementary Figure 1. Notably, ACE2 expression was significantly related to the prognosis of six tumor types (Figure 2): brain, breast, eye, lung, and ovarian cancers and renal cell carcinoma. ACE2 had a protective effect in five types of cancer: brain (OS: total number = 74, HR = 0.44, Cox *P* = 0.003507), eye (*distant metastasis-free survival*: total number = 63, HR = 0.00, Cox *P* = 0.033962), lung (OS: total number = 129, HR = 0.70, Cox *P* = 0.009280), ovarian (OS: total number = 278, HR = 0.63, Cox *P* = 0.048512), and renal (OS: total number = 59, HR = 0.17, Cox *P* = 0.021041). ACE2 only had a detrimental role in breast cancer (RFS: total number = 87, HR = 3.36, Cox *P* = 0.019817; distant metastasis-free survival: total number = 125, HR = 1.55, Cox *P* = 0.038243; OS: total number = 198, HR = 1.23, Cox *P* = 0.002524).

**FIGURE 3:**
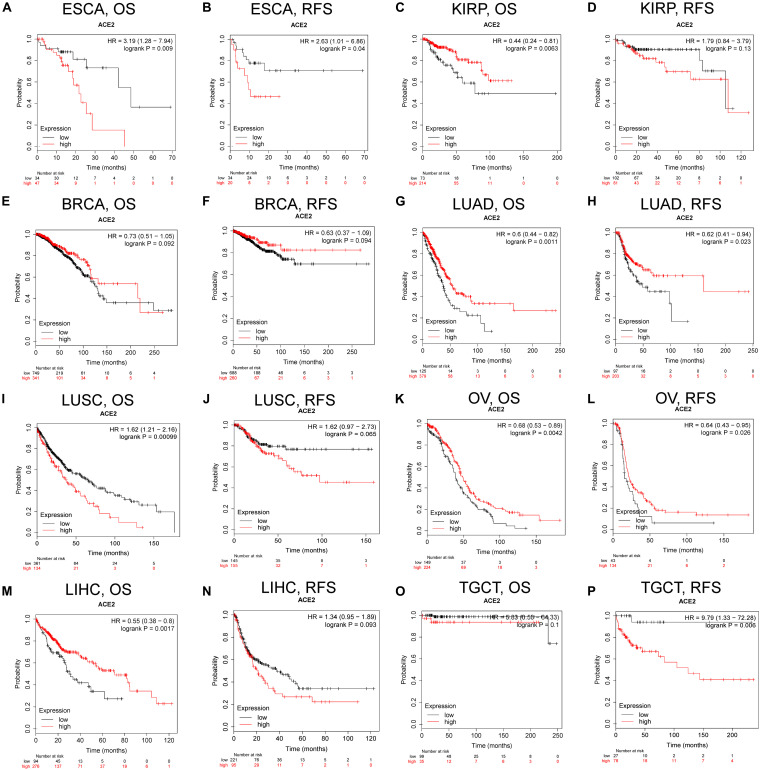
Kaplan–Meier survival curves comparing the high and low expression of ACE2 in different types of cancer in Kaplan–Meier Plotter. OS and RFS of **(A,B)** esophageal carcinoma (ESCA), **(C,D)** kidney renal papillary cell carcinoma (KIRP), **(E,F)** BRCA, **(G,H)** LUAD, **(I,J)** LUSC, **(K,L)** OV, **(M,N)** LIHC, and **(O,P)** TGCTs. Red curve represents patients with high expression of ACE2. OS, overall survival; RFS, relapse-free survival.

Next, we explored the prognostic survival of ACE2-related cancers in Kaplan–Meier Plotter, which uses mainly TCGA data (in contrast to PrognoScan, whose data mainly comes from the Gene Expression Omnibus database). We showed for the first time that ACE2 is related to poor prognosis in esophageal carcinoma (OS: HR = 3.19, log-rank *P* = 0.009; RFS: HR = 2.63, log-rank *P* = 0.04) (Figures 3A,B). The results for kidney cancer and lung cancer were partially different from those obtained in PrognoScan: ACE2 had a positive impact on OS (HR = 0.44, log-rank *P* = 0.0063) but not on RFS (HR = 1.79, log-rank *P* = 0.13) in KIRP (Figures 3C,D); ACE2 was protective in LUAD (OS: HR = 0.6, log-rank *P* = 0.0011; RFS: HR = 0.62, log-rank *P* = 0.023) (Figures 3G,H) but detrimental in LUSC (OS: HR = 1.62, log-rank *P* = 0.00099; RFS: HR = 1.62, log-rank *P* = 0.065) (Figures 3I,J). In ovarian cancer, ACE2 was a protective prognostic factor (OS: HR = 0.68, log-rank *P* = 0.0042; RFS: HR = 0.64, log-rank *P* = 0.026) (Figures 3K,L). In LIHC, ACE2 was beneficial to OS (HR = 0.55, log-rank *P* = 0.0017) but had no effect on RFS (HR = 1.34, log-rank *P* = 0.093) (Figures 3M,N), whereas the opposite was true in testicular germ cell tumor (TGCT) (OS: HR = 5.83, log-rank *P* = 0.1; RFS: HR = 9.79, log-rank *P* = 0.006) (Figures 3O,P).

In addition to PrognoScan and Kaplan–Meier Plotter, we used GEPIA to investigate the role of ACE2 in each cancer and its overall impact on cancer. In general, ACE2 was a protective index for cancer prognosis (OS: total number = 9,478, HR = 1.1, log-rank *P* = 0.14; DFS: total number = 9,478, HR = 0.82, log-rank *P* = 2.2e-07). We found that ACE2 had a protective role in LIHC. The results for kidney cancer were slightly different from those obtained with PrognoScan and Kaplan–Meier Plotter; high expression levels of ACE2 were beneficial in kidney renal clear cell carcinoma (KIRC) but did not affect KICH or KIRP. In ovarian cancer, ACE2 not only had a protective role for OS but was also beneficial to DFS. In LUSC, ACE2 has a protective effect on DFS but no significant effect on OS. We found similar effects in UCS. In LGG, ACE2 had a deleterious effect on OS, contrary to the conclusions obtained using PrognoScan. We did not find any effects of ACE2 on BRCA, head–neck squamous cell carcinoma, PRAD, STAD, or rectum adenocarcinoma (Supplementary Figure 2).

### Angiotensin-Converting Enzyme 2 Expression in a Stratified Population

To better understand the impact of ACE2 on the survival of cancer patients, we investigated the relationship between ACE2 expression and clinical characteristics of LIHC patients using the Kaplan–Meier Plotter database (Table 1). ACE2 was associated with improved OS in many patients with liver cancer, except for those with stage 2 (HR = 0.65, *P* = 0.2872), grade 3 (HR = 0.59, *P* = 0.0839), or American Joint Committee on Cancer stage T2 (HR = 0.71, *P* = 0.3515) disease, or microvascular invasion (HR = 0.5, *P* = 0.1322). In terms of progression-free survival, ACE2 had no benefit in stage 2 (HR = 1.48, *P* = 0.2686) or American Joint Committee on Cancer stage T2 (HR = 1.58, *P* = 0.1717) patients, those with microvascular invasion (HR = 0.78, *P* = 0.4074), female patients (HR = 0.69, *P* = 0.1531), or those undergoing sorafenib treatment (HR = 0.73, *P* = 0.4354). In addition, compared with vascular invasion, the HR values for OS and progression-free survival in patients without vascular invasion showed statistically significant differences. These results indicate that high expression levels of ACE2 may improve survival in patients with no vascular invasion.

**TABLE 1:** Correlation of ACE2 messenger RNA expression with OS (*n* = 364) and PFS (*n* = 366) in LIHC with different clinicopathological features.

Clinicopathological features	OS (*n* = 364)			PFS (*n* = 366)		
	*N*	Hazard ratio	*P*	*N*	Hazard ratio	*P*
**Stage**						
1	170	0.32(0.15–0.67)	0.0015	170	0.4(0.24–0.66)	0.0002
1 + 2	253	0.48(0.27–0.82)	0.0067	254	0.57(0.39–0.83)	0.0031
2	83	0.65(0.3–1.44)	0.2872	84	1.48(0.74–2.95)	0.2686
2 + 3	166	0.56(0.35–0.9)	0.0148	167	0.63(0.41–0.96)	0.0298
3	83	0.38(0.2–0.72)	0.002	83	0.42(0.23–0.75)	0.0029
3 + 4	87	0.42(0.23–0.77)	0.0039	88	0.44(0.25–0.79)	0.0045
4	4	–	–	5	–	–
**Grade**						
1	55	0.27(0.1–0.7)	0.0042	55	0.4(0.18–0.87)	0.0173
2	174	0.56(0.34–0.93)	0.0228	175	0.54(0.35–0.84)	0.0056
3	118	0.59(0.32–1.08)	0.0839	119	0.6(0.36–0.99)	0.0429
4	12	–	–	12	–	–
**AJCC_T**						
1	180	0.31(0.15–0.63)	0.0006	180	0.37(0.22–0.6)	3.10E−05
2	90	0.71(0.34–1.47)	0.3515	92	1.58(0.82–3.04)	0.1717
3	78	0.34(0.17–0.66)	0.0009	78	0.31(0.16–0.6)	0.0002
4	13	–	–	13	–	–
**Vascular invasion**						
None	203	0.55(0.32–0.93)	0.0236	204	0.49(0.31–0.76)	0.0013
Micro	93	0.5(0.2–1.25)	0.1322	91	0.78(0.43–1.4)	0.4074
Macro	16	–	–	16	–	–
Sex						
Male	246	0.51(0.31–0.81)	0.004	246	0.5(0.35–0.71)	0.0001
Female	118	0.43(0.24–0.75)	0.0021	120	0.69(0.41–1.15)	0.1531
Race						
White	181	0.54(0.34–0.86)	0.0091	183	0.69(0.45–1.05)	0.0788
Black of African American	17	–	–	17	–	–
Asian	155	0.26(0.1–0.68)	0.003	155	0.35(0.22–0.56)	6.70E−06
**Sorafenib treatment**						
Treated	29	0.27(0.09–0.86)	0.0174	30	0.73(0.33–1.61)	0.4354
**Alcohol consumption**						
Yes	115	0.42(0.21–0.82)	0.0095	115	0.4(0.23–0.67)	0.0004
None	202	0.42(0.26–0.69)	0.0004	204	0.55(0.37–0.83)	0.0042
**Hepatitis virus**						
Yes	150	0.51(0.26–1)	0.0472	152	0.49(0.31–0.78)	0.0021
None	167	0.41(0.26–0.66)	0.0001	167	0.54(0.35–0.84)	0.0057

*OS, overall survival; PFS, progression-free survival.*

### Contradictory Results for Correlation of Angiotensin-Converting Enzyme 2 Expression With Immune Infiltration

**FIGURE 4:**
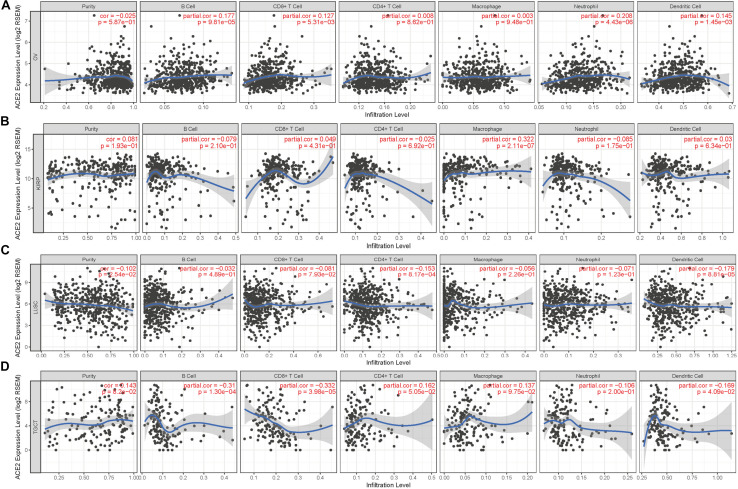
Correlation of ACE2 expression with immune infiltration level. **(A)** ACE2 expression has no relation with tumor purity and significant positive correlation with infiltrating levels of B cell, CD8 + T cell, neutrophil, and dendritic cell in OV. **(B)** ACE2 expression has no relation with tumor purity and significant positive correlation with infiltrating levels of macrophage in KIRP. **(C)** ACE2 expression has significant positive correlation with tumor purity, significant negative correlation with infiltrating levels of CD4 + T cell and dendritic cell in LUSC. **(D)** ACE2 expression has no relation with tumor purity and significant negative correlation with infiltrating levels of B cell, CD8 + T cell, and dendritic cell in TGCT. *P* < 0.05 is considered as significant.

The discussed results support a pan-cancer prognostic role of ACE2 and indicate that immune cells in the TME can positively influence the survival of patients. Therefore, we explored the association between immune infiltration and ACE2 expression. We calculated the coefficients for the correlation between ACE2 expression and immune infiltration levels in 39 cancer types via the TIMER database. The results showed that ACE2 expression was significantly correlated with tumor purity in 11 cancer types. Also, expression levels of ACE2 were significantly correlated with infiltration levels of B cells, CD8 + T cells, CD4 + T cells, macrophages, neutrophils, and dendritic cells in 13, 11, 5, 10, 16, and 15 cancer types (Figure 4 and Supplementary Figure 3).

According to the findings from Kaplan–Meier Plotter, GEPIA, and PrognoScan, we observed that it is consistent with the immune infiltration level in TIMER. Among the cancer types in which ACE2 has a protective role, expression levels of ACE2 were significantly positively correlated with infiltration levels of B cells (*R* = 0.177, *P* = 9.81E-05), CD8 + T cells (*R* = 0.127, *P* = 5.31E-03), neutrophils (*R* = 0.208, *P* = 4.43E-06), and dendritic cells (*R* = 0.145, *P* = 1.45E-03) in OV (Figure 4A); macrophages (*R* = 0.322, *P* = 2.11E-07) in KIRP (Figure 4B); B cells (*R* = 0.194, *P* = 2.97E-05) and CD8 + T cells (*R* = 0.097, *P* = 4.31E-02) in KIRC (Supplementary Figure 3A); and B cells (*R* = 0.166, *P* = 4.55E-03), CD4 + T cells (*R* = 0.154, *P* = 8.56E-03), neutrophils (*R* = 0.223, *P* = 1.18E-04), and dendritic cells (*R* = 0.145, *P* = 2.60E-06) in UCEC (Supplementary Figure 3B). In cancer types where ACE2 was a deleterious prognostic factor, the infiltration level of various immune cells was negatively correlated with the expression level of ACE2: CD4 + T cells (*R* = −0.153, *P* = 8.17E-04) and dendritic cells (*R* = −0.179, *P* = 8.81E-05) in LUSC (Figure 4C); B cells (*R* = −0.31, *P* = 1.30E-04), CD8 + T cells (*R* = −0.332, *P* = 3.98E-05), and dendritic cells (*R* = −0.169, *P* = 4.09E-02) in TGCT (Figure 4D); B cells (*R* = −0.117, *P* = 1.08E-02) and dendritic cells (*R* = −0.097, *P* = 3.49E-02) in LGG (Supplementary Figure 3C); and B cells (*R* = −0.238, *P* = 1.07E-02), CD4 + T cells (*R* = −0.258, *P* = 6.35E-03), macrophages (*R* = −0.348, *P* = 1.46E-04), and neutrophils (*R* = −0.329, P = 3.55E-04) in t*hymomaTHYM* (Supplementary Figure 3D). In addition, the expression of ACE2 in PRAD (Supplementary Figure 3E) was positively correlated with the infiltration level of each immune cell type, but we did not find a significant correlation between ACE2 and survival. The discussed results suggest that ACE2 could affect the prognosis and survival of patients via immune infiltration.

### Relationships Between Angiotensin-Converting Enzyme 2 Expression and Immune Markers

To further explore the correlation between ACE2 and immune cell infiltration, we investigated the relationships between ACE2 and immune cell markers in the TIMER database, including B cells, CD8 + T cells, M1/M2 macrophages, tumor-associated macrophages, monocytes, NK cells, neutrophils, and dendritic cells. We also analyzed various subtypes of T cells, including follicular helper T, Th1, Th2, Th9, Th17, Th22, regulatory T cells, and exhausted T cells. After correction of tumor purity, expression levels of ACE2 were significantly correlated with 21, 22, 34, 25, 23, and 30 immune cell markers in KIRP, KIRC, LIHC, LUSC, OV, and TGCT, respectively (Tables 2, 3 and Supplementary Table 1). These results are consistent with those described earlier, concerning the relationships between various types of cancer and the infiltration of immune cells. Therefore, these results confirm our conjecture that the prognosis of ACE2-related tumors is significantly correlated with immune cell infiltration, which helps explain the discrepancies in patient survival.

**TABLE 2:** Correlations between ACE2 and gene markers of immune cells in LIHC and KIPR.

Gene type	Gene marker	LIHC				KIRP			
		None		Purity		None		Purity	
		Cor	*P*	Cor	*P*	Cor	*P*	Cor	*P*
B cell	CD19	–0.082	0.116	–0.101	0.061	–0.058	0.325	–0.032	0.612
	CD20	–0.101	0.052	–0.128	0.017	0.029	0.629	0.045	0.475
	CD38	–0.157	*	–0.205	**	–0.060	0.307	–0.092	0.140
CD8 + T cell	CD8A	–0.081	0.118	–0.117	0.030	0.031	0.594	0.021	0.738
	CD8B	–0.076	0.142	–0.104	0.053	0.017	0.771	0.014	0.829
Tfh	CXCR5	–0.163	*	–0.195	**	–0.071	0.226	–0.080	0.202
	ICOS	–0.231	***	–0.290	***	0.005	0.931	0.004	0.949
	BCL6	0.084	0.107	0.105	0.051	–0.222	**	–0.244	***
Th1	IL12RB2	0.179	**	0.164	*	0.240	***	0.204	*
	IL27RA	–0.157	*	–0.188	**	–0.175	*	–0.176	*
	T-bet	–0.019	0.710	–0.042	0.434	0.048	0.414	0.051	0.410
Th2	CCR3	–0.004	0.935	–0.008	0.882	–0.076	0.195	–0.097	0.119
	STAT6	0.199	**	0.208	**	0.242	***	0.249	***
	GATA3	–0.087	0.095	–0.108	0.045	–0.277	***	–0.250	***
Th9	TGFBR2	0.269	***	0.279	***	–0.113	0.055	–0.144	0.021
	IRF4	–0.128	0.013	–0.166	*	–0.117	0.047	–0.136	0.029
	PU.1	–0.143	*	–0.233	***	0.092	0.117	0.093	0.136
Th17	IL21R	–0.143	*	–0.198	**	–0.112	0.057	–0.113	0.070
	IL23R	0.096	0.065	0.117	0.030	0.056	0.346	0.071	0.255
	RORC	0.220	***	0.247	***	–0.123	0.036	–0.162	*
	STAT3	0.106	0.042	0.091	0.092	–0.100	0.089	–0.133	0.033
Th22	CCR10	–0.140	*	–0.147	*	–0.325	***	–0.317	***
	AHR	0.184	**	0.197	**	–0.076	0.199	–0.092	0.143
Treg	FOXP3	0.063	0.225	0.039	0.475	–0.144	0.014	–0.145	0.019
	CCR8	–0.074	0.154	–0.095	0.077	–0.049	0.403	–0.074	0.233
	CD25	–0.166	*	–0.237	***	–0.087	0.137	–0.103	0.097
T cell exhaustion	PD-1	–0.218	***	–0.271	***	–0.039	0.506	–0.032	0.611
	TIM-3	–0.122	0.019	–0.192	**	0.398	***	0.401	***
	LAG3	–0.098	0.059	–0.119	0.027	–0.091	0.122	–0.095	0.127
	CTLA4	–0.266	***	–0.318	***	–0.080	0.176	–0.092	0.142
Macrophage	CD68	–0.138	*	–0.172	*	0.339	***	0.323	***
	CD11b	–0.063	0.229	–0.105	0.051	0.077	0.191	0.078	0.211
M1	NOS2	0.233	***	0.216	***	–0.092	0.117	–0.129	0.039
	CD86	–0.122	0.019	–0.195	**	0.139	0.018	0.134	0.032
	CD80	–0.106	0.041	–0.175	*	0.047	0.429	0.038	0.539
	ROS	0.191	**	0.192	**	–0.256	***	–0.262	***
M2	ARG1	0.105	0.042	0.101	0.060	–0.058	0.326	–0.045	0.473
	CD163	0.040	0.447	0.010	0.848	0.155	*	0.161	*
TAM	HLA-G	0.041	0.434	0.014	0.797	0.209	**	0.238	**
Monocyte	CD14	0.060	0.249	0.066	0.222	0.082	0.164	0.091	0.146
	CD16B	0.105	0.044	0.091	0.091	–0.022	0.707	–0.025	0.692
NK	XCL1	–0.204	***	–0.218	***	–0.013	0.832	–0.011	0.862
	CD56	0.060	0.249	0.052	0.332	–0.250	***	–0.235	**
	KIR3DL1	0.134	*	0.119	0.027	0.049	0.409	0.030	0.632
	CD7	–0.198	**	–0.251	***	–0.096	0.102	–0.107	0.088
Neutrophil	CD15	–0.123	0.018	–0.142	*	0.125	0.033	0.122	0.050
DC	CD1C	–0.029	0.581	–0.049	0.364	–0.040	0.500	–0.053	0.395
	CD141	0.138	*	0.145	*	–0.213	**	–0.216	**
	CLEC9A	–0.030	0.568	–0.033	0.541	0.140	0.017	0.141	0.024
Breg	CD1d	0.160	*	0.159	*	–0.071	0.230	–0.099	0.112
	CD5	–0.111	0.033	–0.157	*	0.055	0.348	0.057	0.358

LIHC, liver hepatocellular carcinoma; KIRP, kidney renal papillary cell carcinoma; Tfh, follicular helper T cell; Th, T helper cell; Treg, regulatory T cell; TAM, tumor-associated- macrophage; NK, natural killer cell; DC, dendritic cell; Breg, regulatory B cell; Purity, correlation adjusted for tumor purity; Cor, *R*-value of Spearman’s correlation. *P < 0.01; **P < 0.001; ***P < 0.0001.

**TABLE 3:** Correlations between ACE2 and gene markers of immune cells in OV and TGCT.

Gene type	Gene marker	OV				TGCT			
		None		Purity		None		Purity	
		Cor	*P*	Cor	*P*	Cor	*P*	Cor	*P*
B cell	CD19	0.043	0.457	0.031	0.624	–0.259	*	–0.214	*
	CD20	0.035	0.549	0.021	0.747	–0.167	0.042	–0.082	0.326
	CD38	0.322	***	0.348	***	–0.375	***	–0.353	***
CD8 + T cell	CD8A	0.067	0.248	0.069	0.277	–0.173	0.034	–0.095	0.252
	CD8B	–0.021	0.711	–0.049	0.443	–0.040	0.627	0.060	0.470
Tfh	CXCR5	0.158	*	0.140	0.027	–0.116	0.157	–0.019	0.824
	ICOS	0.183	*	0.194	*	–0.132	0.108	–0.033	0.692
	BCL6	0.070	0.225	0.105	0.099	0.359	***	0.363	***
Th1	IL12RB2	0.130	0.024	0.113	0.076	–0.180	0.028	–0.121	0.143
	IL27RA	0.029	0.617	0.028	0.657	0.148	0.072	0.195	0.018
	T-bet	0.142	0.013	0.161	0.011	–0.271	**	–0.227	*
Th2	CCR3	0.240	***	0.287	***	0.178	0.030	0.213	*
	STAT6	0.206	**	0.193	*	0.520	***	0.511	***
	GATA3	0.032	0.582	0.032	0.610	0.359	***	0.350	***
Th9	TGFBR2	0.045	0.434	0.041	0.520	0.409	***	0.403	***
	IRF4	0.061	0.290	0.046	0.469	–0.232	*	–0.174	0.035
	PU.1	0.061	0.292	0.074	0.246	–0.126	0.125	–0.039	0.639
Th17	IL21R	0.032	0.574	0.016	0.803	–0.197	0.016	–0.119	0.151
	IL23R	0.140	0.015	0.160	0.011	–0.124	0.129	–0.091	0.275
	RORC	0.111	0.054	0.096	0.132	0.297	**	0.337	***
	STAT3	0.096	0.094	0.113	0.076	0.380	***	0.380	***
Th22	CCR10	–0.158	*	–0.107	0.092	0.448	***	0.433	***
AHR		0.184	0.105	0.068	0.109	0.086	0.318	*⁣*⁣*	0.328
Treg	FOXP3	0.128	0.026	0.145	0.022	–0.146	0.074	–0.058	0.483
	CCR8	0.101	0.079	0.081	0.205	–0.056	0.492	–0.019	0.815
	CD25	0.009	0.872	0.044	0.486	–0.152	0.064	–0.066	0.428
T cell exhaustion	PD-1	0.112	0.052	0.146	0.021	–0.282	**	–0.243	*
	TIM-3	0.116	0.044	0.143	0.024	–0.101	0.221	0.000	0.996
	LAG3	0.258	***	0.311	***	–0.300	**	–0.267	*
	CTLA4	0.152	*	0.195	*	–0.250	*	–0.193	0.019
Macrophage	CD68	0.098	0.088	0.119	0.060	–0.023	0.780	0.073	0.378
	CD11b	0.067	0.244	0.059	0.357	–0.031	0.702	0.043	0.609
M1	NOS2	0.080	0.165	0.127	0.046	0.385	***	0.365	***
	CD86	0.141	0.014	0.174	*	–0.133	0.104	–0.041	0.625
	CD80	0.216	**	0.255	***	–0.181	0.027	–0.111	0.180
	ROS	0.070	0.222	0.094	0.141	0.482	***	0.452	***
M2	ARG1	–0.003	0.957	–0.023	0.716	0.211	*	0.169	0.041
	CD163	0.094	0.104	0.116	0.068	0.281	**	0.322	***
TAM	HLA-G	0.229	***	0.221	**	0.213	*	0.232	*
Monocyte	CD14	0.023	0.690	0.040	0.529	0.150	0.066	0.220	*
	CD16B	0.147	0.011	0.155	0.014	0.252	*	0.248	*
NK	XCL1	–0.054	0.345	–0.043	0.496	–0.039	0.639	0.017	0.842
	CD56	–0.108	0.059	–0.071	0.264	0.512	***	0.499	***
	KIR3DL1	0.138	0.016	0.166	*	–0.157	0.054	–0.077	0.355
	CD7	0.120	0.036	0.145	0.022	–0.174	0.034	–0.099	0.231
Neutrophil	CD15	0.248	***	0.250	***	–0.127	0.123	–0.068	0.411
DC	CD1C	–0.078	0.178	–0.131	0.039	0.276	**	0.330	***
	CD141	0.009	0.882	0.018	0.776	0.639	***	0.627	***
	CLEC9A	0.092	0.111	0.126	0.048	–0.355	***	–0.318	***
Breg	CD1d	0.051	0.372	0.014	0.823	0.016	0.843	0.077	0.355
	CD5	0.107	0.064	0.108	0.090	–0.132	0.107	–0.035	0.671

OV, ovarian serous cystadenocarcinoma; TGCTs, testicular germ cell tumors; Tfh, follicular helper T cell; Th, T helper cell; Treg, regulatory T cell; TAM, tumor-associated- macrophage; NK, natural killer cell; DC, dendritic cell; Breg, regulatory B cell; Purity, correlation adjusted for tumor purity; Cor, *R*-value of Spearman’s correlation. *P < 0.01; **P < 0.001; ***P < 0.0001.

## Discussion

ACE2 has been implicated in cardiac dysfunction, hypertension (Crackower et al., 2002), heart failure and ventricular remodeling (Donoghue et al., 2003; Zisman et al., 2003), diabetes (Tikellis et al., 2003), Ang 1–7 regulation during pregnancy (Brosnihan et al., 2003), and as a functional receptor for coronaviruses, including SARS-CoV and SARS-CoV-2 (Li et al., 2003; Zhou et al., 2020). Recently, researchers have found that members of the RAS participate in various biological processes in tumors. AngII has been reported to facilitate tumor migration, proliferation, angiogenesis, and metastasis by activating the AngII type 1 receptor, whereas activation of the AngII type 2 receptor promotes tumor proliferation and angiogenesis in lung cancer (Kanehira et al., 2005; Pickel et al., 2010). ACE2, as well as Ang 1–7, has been reported to inhibit the growth of lung cancer (Menon et al., 2007; Ni et al., 2012; Qian et al., 2013) and metastasis of prostate cancer (Krishnan et al., 2013b), and it is associated with better prognosis in hepatocellular carcinoma (Ye et al., 2015).

Although the relationship between the expression of ACE2 and the proliferation and progression of certain tumors has been confirmed at the molecular level and in animal models, in-depth research has not been conducted for clinical diagnosis and treatment. As previous studies and the current results indicate, ACE2 may have different roles in different situations; thus, it is necessary to explore the correlations between ACE2 and cancer from a clinical perspective. In this study, we found that the prognosis of ACE2 and cancer may differ owing to characteristics such as sex, race, and tumor stage. More comprehensive and precise research will be required in the future to find a reasonable explanation for these differences. Understanding the TME, including the infiltration of immune cells, can help reveal the important mechanisms underlying tumor development. Here, we found a significant correlation between tumor ACE2 expression and immune cell infiltration. Several studies have revealed that ACE2 intervenes in the progression and metastasis of tumors via inhibiting tumor cell proliferation, invasion, migration, and angiogenesis through the ACE2/Ang 1–7/MasR axis (Xu et al., 2017; de Paula et al., 2020); this has been demonstrated in hepatocellular carcinoma (Liu et al., 2015; Mao et al., 2018), prostate cancer (Krishnan et al., 2013a, b; Dominska et al., 2018), nasopharyngeal cancer (Pei et al., 2016; Lin et al., 2018), and head and neck cancer (Hinsley et al., 2017). However, the mechanism by which ACE2 affects tumors via the regulation of the immune microenvironment is not yet clear; this is a potential direction for our future research.

This study explored the expression levels of ACE2 and visualized the prognostic landscape on a pan-cancer basis using independent datasets in Oncomine and PrognoScan, and TCGA data in GEPIA and TIMER. Based on the analysis of these four databases, we concluded that ACE2 was beneficial in KIRP, LUAD, OV, and LIHC but had a detrimental effect on prognosis in esophageal carcinoma, LUSC, TGCT, and LGG. Compared with normal tissues, expression levels of ACE2 in BRCA, KICH, LIHC, and PRAD were significantly reduced, and those of KIRP, LUAD, and UCEC were significantly increased. However, the expression of ACE2 was not related to tumor purity in cancers with good prognosis, such as KIRC, KIRP, LIHC, OV, and LUAD. These findings suggest that ACE2 is equally expressed in tumor cells and the TME.

We also found relationships among ACE2 expression, tumor prognosis, and immune cell infiltration. ACE2 expression levels in tumors with a good prognosis, such as KIRP, LIHC, and OV, were significantly positively correlated with infiltration of immune cells, including B cells, CD8 + T cells, CD4 + T cells, neutrophils, macrophages, and dendritic cells, whereas they were significantly negatively correlated with infiltration in tumors with poor prognoses, such as LUSC and TGCT. This suggests that ACE2 may affect tumor prognosis through cancer immunity. We further observed that ACE2 was most strongly related to the expression of B cells, T cells, and dendritic cells. Together with a previous study confirming that ACE2 is involved in the immune process (Cui et al., 2017), these results indicate that ACE2 may play an important part in tumor antigen presentation and tumor killing.

Although we integrated information from multiple databases, this study had some limitations. Given that a large proportion of gene chip and sequencing data were collected by analysis of tumor tissue information, systematic bias may have been introduced into the cell-level analysis of immune cell markers. To solve this problem, higher-resolution methods should be used in the future, such as single-cell RNA sequencing (Giladi and Amit, 2018; Papalexi and Satija, 2018). Moreover, as there was only one LIHC dataset containing complete clinicopathological characteristics, the results obtained were not comprehensive. More clinical data will need to be investigated in the future to clarify the relationship between ACE2 and clinical characteristics of cancer patients and, possibly, between coronavirus disease of 2019 and cancer. Owing to contradictions between different databases, we could not clearly define ACE2 as either beneficial or detrimental to tumors. Furthermore, this study only involved bioinformatics analysis of ACE2 expression and patient survival; no *in vivo* or *in vitro* experiments were performed. Such experiments will be needed to clarify the specific mechanism of the action of ACE2. It is also worth noting that we have explored the correlation between the expression of ACE2 and the infiltration of tumor immune cells. Although the expression of ACE2 is certainly related to its enzymatic activity, expression of an enzyme does not necessarily reflect activity. We will conduct relevant functional experiments in our next study to verify the correlation between the ACE2 enzyme and tumor immune cell infiltration. Finally, although studies have found that ACE2 expression is related to tumor immune cell infiltration and patient survival, we could not directly show whether ACE2 affects patient survival via immune cell infiltration. Future prospective studies on ACE2 expression and immune cell infiltration in cancer populations will help to provide a clear answer to this question. In summary, ACE2 can affect pan-cancer prognosis and is related to immune infiltration and could be used as a pan-cancer prognostic biomarker. These findings may inform immune-based antitumor strategies for clinical treatments, including immune cell infiltration of tumor cells or the TME.

## Data Availability Statement

Publicly available datasets were analyzed in this study. This data can be found here: “Oncomine (www.oncomine.org), PrognoScan (http://dna00.bio.kyutech.ac.jp/PrognoScan/index.html), Kaplan–Meier Plotter (https://kmplot.com/analysis/), GEPIA (http://gepia.cancer-pku.cn), and TIMER (http://cistrome.org/TIMER/)”.

## Author Contributions

HF and XW designed this study and analyzed the data. LP, YW, BH, YR, and ZL extracted the information from the databases. HF drafted the manuscript. TW revised the manuscript. JL and TW supervised the entire study. All authors read and approved the final manuscript.

## Conflict of Interest

The authors declare that the research was conducted in the absence of any commercial or financial relationships that could be construed as a potential conflict of interest.

## Footnotes

1 http://www.oncomine.org

2 http://dna00.bio.kyutech.ac.jp/PrognoScan/index.html

3 https://kmplot.com/analysis/

4 http://gepia.cancer-pku.cn/

5 www.r-project.org

6 http://cistrome.shinyapps.io/timer

7 https://www.cst-c.com.cn/common/content/content.jsp?id=pathways-ti-icm-human

8 https://www.rndsystems.com/cn/resources/cell-markers/immune-cells

## Abbreviations

ACE2: angiotensin-converting enzyme 2
BRCA: breast invasive carcinoma
CESC: cervical squamous cell carcinoma and endocervical adenocarcinoma
DFS: disease-free survival
DSS: disease-specific survival
EFS: event-free survival
GBM: glioblastoma multiforme
KICH: kidney chromophobe
KIRP: kidney renal papillary cell carcinoma
LGG: brain lower grade glioma
LIHC: liver hepatocellular carcinoma
LUAD: lung adenocarcinoma
LUSC: lung squamous cell carcinoma
OS: overall survival
OV: ovarian serous cystadenocarcinoma
PRAD: prostate adenocarcinoma
RFS: relapse-free survival
STAD: stomach adenocarcinoma
THCA: thyroid carcinoma
UCEC: uterine corpus endometrial carcinoma.
