# Supplementary material for: Prognostic and Immunological Value of Angiotensin-Converting Enzyme 2 in Pan-Cancer
Source: Front Mol Biosci. 2020 Sep 1;7:189. doi: 10.3389/fmolb.2020.00189 (PMC7490340; doi:10.3389/fmolb.2020.00189)
Supplement: Supplementary file 1 [file Data_Sheet_1.ZIP › Supplementary Figures legends.docx]

**Supplementary Figure Legends**

**Supplementary Figure 1.** Relation between ACE2 expression and patient prognosis of different datasets of cancers in PrognoScan. Red square represents hazard ratio. OS, overall survival; DSS, disease-specific survival; EFS, event-free survival; DMFS, distant metastasis-free survival; RFS, relapse-free survival; DFS, disease-free survival.

**Supplementary Figure 2.** Correlation of ACE2 expression with prognostic values in cancers. Overall survival and disease free survival of **(A)** all cancer types **(B)** ACC, Adrenocortical carcinoma **(C)** BLCA, Bladder Urothelial Carcinoma **(D)** BRCA, Breast invasive carcinoma **(E)** CESC, Cervical squamous cell carcinoma and endocervical adenocarcinoma **(F)** CHOL, Cholangio carcinoma **(G)** COAD, Colon adenocarcinoma **(H)** ESCA, Esophageal carcinoma **(I)** GBM, Glioblastoma multiforme **(J)** HNSC, Head and Neck squamous cell carcinoma **(K)** KICH, Kidney Chromophobe **(L)** KIRC, Kidney renal clear cell carcinoma **(M)** KIRP, Kidney renal papillary cell carcinoma **(N)** LGG, Brain Lower Grade Glioma **(O)** LIHC, Liver hepatocellular carcinoma **(P)** LUAD, Lung adenocarcinoma **(Q)** LUSC, Lung squamous cell carcinoma **(R)** OV, Ovarian serous cystadenocarcinoma **(S)** PAAD, Pancreatic adenocarcinoma **(T)** PCPG, Pheochromocytoma and Paraganglioma **(U)** PRAD, Prostate adenocarcinoma **(V)** READ, Rectum adenocarcinoma **(W)** SARC, Sarcoma **(X)** STAD, Stomach adenocarcinoma **(Y)** TGCT, Testicular Germ Cell Tumors **(Z)** THCA, Thyroid carcinoma **(AA)** THYM, Thymoma **(AB)** UCEC, Uterine Corpus Endometrial Carcinoma **(AC)** UCS, Uterine Carcinosarcoma and **(AD)** UVM, Uveal Melanoma.

**Supplementary Figure 3.** Correlation of ACE2 expression with immune infiltration levels in cancers via TIMER database. Correlation of ACE2 expression with immune infiltration levels in **(A)** KIRC, Kidney renal clear cell carcinoma **(B)** UCEC, Uterine Corpus Endometrial Carcinoma **(C)** LGG, Brain Lower Grade Glioma **(D)** THYM, Thymoma **(E)** PRAD, Prostate adenocarcinoma **(F)** ACC, Adrenocortical Carcinoma **(G)** BLCA, Bladder Urothelial Carcinoma **(H)** BRCA-Her2, Breast invasive carcinoma-Her2 **(I)** BRCA-Luminal, Breast invasive carcinoma-Luminal **(J)** BRCA, Breast invasive carcinoma **(K)** CESC, Cervical squamous cell carcinoma and endocervical adenocarcinoma **(L)** CHOL, Cholangio carcinoma **(M)** COAD, Colon adenocarcinoma **(N)** DLBC, Lymphoid Neoplasm Diffuse Large B-cell Lymphoma **(O)** ESCA, Esophageal carcinoma **(P)** GBM, Glioblastoma multiforme **(Q)** HNSC-HPVneg, , Head and Neck squamous cell carcinoma-HPVneg **(R)** HNSC-HPVpos, Head and Neck squamous cell carcinoma-HPVpos **(S)** HNSC, Head and Neck squamous cell carcinoma **(T)** KICH, Kidney Chromophobe **(U)** LIHC, Liver hepatocellular carcinoma **(V)** LUAD, Lung adenocarcinoma **(W)** MESO, Mesothelioma **(X)** PAAD, Pancreatic adenocarcinoma **(Y)** PCPG, Pheochromocytoma and Paraganglioma **(Z)** READ, Rectum adenocarcinoma **(AA)** SARC, Sarcoma **(AB)** SKCM-Metastasis, Skin Cutaneous Melanoma-Metastasis **(AC)** SKCM-Primary, Skin Cutaneous Melanoma-Primary **(AD)** SKCM, Skin Cutaneous Melanoma **(AE)** STAD, Stomach adenocarcinoma **(AF)** THCA, Thyroid carcinoma **(AG)** UCS, Uterine Carcinosarcoma **(AH)** UVM, Uveal Melanoma.
