# Supplementary material for: Prognostic and Immunological Value of Angiotensin-Converting Enzyme 2 in Pan-Cancer
Source: Front Mol Biosci. 2020 Sep 1;7:189. doi: 10.3389/fmolb.2020.00189 (PMC7490340; doi:10.3389/fmolb.2020.00189)
Supplement: Supplementary file 1 [file Data_Sheet_1.ZIP › Supplementary Table 1.docx]

SUPPLEMENTARY TABLE 1 | Correlations between ACE2 and Gene Markers of Immune Cells in KIRC and LUSC.

| Cell type | Gene marker |  |  |  | KIRC |  |  |  |  |  |  |  | LUSC |  | | |
| --- | --- | --- | --- | --- | --- | --- | --- | --- | --- | --- | --- | --- | --- | --- | --- | --- |
|  |  |  | None |  |  |  | Purity |  |  |  | None |  |  |  | Purity |  |
|  |  | Cor |  | *P* |  | Cor |  | *P* |  | Cor |  | *P* |  | Cor |  | *P* |
| B cell | CD19 | -0.202 |  | *** |  | -0.210 |  | *** |  | 0.017 |  | 0.698 |  | -0.019 |  | 0.674 |
|  | CD20 | -0.076 |  | 0.081 |  | -0.083 |  | 0.075 |  | 0.035 |  | 0.430 |  | -0.002 |  | 0.969 |
|  | CD38 | 0.048 |  | 0.264 |  | 0.018 |  | 0.694 |  | 0.036 |  | 0.426 |  | 0.014 |  | 0.761 |
| CD8+ T Cell | CD8A | 0.069 |  | 0.111 |  | 0.058 |  | 0.213 |  | -0.040 |  | 0.375 |  | -0.076 |  | 0.097 |
|  | CD8B | 0.077 |  | 0.076 |  | 0.065 |  | 0.165 |  | -0.104 |  | 0.020 |  | -0.135 |  | * |
| Tfh | CXCR5 | -0.214 |  | *** |  | -0.219 |  | *** |  | -0.026 |  | 0.565 |  | -0.076 |  | 0.099 |
|  | ICOS | 0.045 |  | 0.302 |  | 0.022 |  | 0.635 |  | -0.043 |  | 0.342 |  | -0.097 |  | 0.034 |
|  | BCL6 | -0.278 |  | *** |  | -0.304 |  | *** |  | 0.259 |  | *** |  | 0.277 |  | *** |
| Th1 | IL12RB2 | 0.130 |  | * |  | 0.113 |  | 0.015 |  | 0.010 |  | 0.830 |  | -0.007 |  | 0.877 |
|  | IL27RA | -0.251 |  | *** |  | -0.280 |  | *** |  | -0.150 |  | ** |  | -0.174 |  | ** |
|  | T-bet | -0.014 |  | 0.743 |  | -0.046 |  | 0.323 |  | -0.033 |  | 0.456 |  | -0.070 |  | 0.125 |
| Th2 | CCR3 | 0.029 |  | 0.501 |  | 0.049 |  | 0.298 |  | 0.094 |  | 0.036 |  | 0.070 |  | 0.124 |
|  | STAT6 | 0.084 |  | 0.054 |  | 0.073 |  | 0.119 |  | 0.204 |  | *** |  | 0.202 |  | *** |
|  | GATA3 | -0.293 |  | *** |  | -0.253 |  | *** |  | -0.086 |  | 0.054 |  | -0.118 |  | 0.010 |
| Th9 | TGFBR2 | 0.150 |  | ** |  | 0.113 |  | 0.015 |  | 0.009 |  | 0.846 |  | -0.031 |  | 0.493 |
|  | IRF4 | -0.043 |  | 0.318 |  | -0.065 |  | 0.161 |  | 0.009 |  | 0.833 |  | -0.032 |  | 0.487 |
|  | PU.1 | -0.111 |  | 0.010 |  | -0.118 |  | 0.011 |  | -0.083 |  | 0.064 |  | -0.152 |  | ** |
| Th17 | IL21R | -0.210 |  | *** |  | -0.219 |  | *** |  | -0.090 |  | 0.044 |  | -0.145 |  | * |
|  | IL23R | 0.019 |  | 0.655 |  | 0.029 |  | 0.528 |  | 0.138 |  | * |  | 0.116 |  | 0.011 |
|  | RORC | 0.265 |  | *** |  | 0.238 |  | *** |  | 0.005 |  | 0.915 |  | -0.016 |  | 0.730 |
|  | STAT3 | -0.037 |  | 0.396 |  | -0.050 |  | 0.286 |  | 0.104 |  | 0.020 |  | 0.096 |  | 0.037 |
| Th22 | CCR10 | -0.326 |  | *** |  | -0.331 |  | *** |  | -0.109 |  | 0.015 |  | -0.128 |  | * |
|  | AHR | 0.074 |  | 0.088 |  | 0.056 |  | 0.230 |  | 0.111 |  | 0.013 |  | 0.108 |  | 0.019 |
| Treg | FOXP3 | -0.165 |  | ** |  | -0.183 |  | *** |  | -0.069 |  | 0.124 |  | -0.120 |  | * |
|  | CCR8 | -0.035 |  | 0.420 |  | -0.052 |  | 0.267 |  | -0.025 |  | 0.570 |  | -0.073 |  | 0.113 |
|  | CD25 | -0.277 |  | *** |  | -0.304 |  | *** |  | -0.039 |  | 0.387 |  | -0.088 |  | 0.054 |
| T cell exhaustion | PD-1 | 0.005 |  | 0.910 |  | -0.013 |  | 0.788 |  | -0.049 |  | 0.271 |  | -0.088 |  | 0.056 |
|  | TIM-3 | 0.289 |  | *** |  | 0.254 |  | *** |  | -0.049 |  | 0.274 |  | -0.101 |  | 0.028 |
|  | LAG3 | -0.021 |  | 0.628 |  | -0.027 |  | 0.564 |  | -0.029 |  | 0.518 |  | -0.061 |  | 0.181 |
|  | CTLA4 | -0.040 |  | 0.355 |  | -0.061 |  | 0.194 |  | -0.071 |  | 0.112 |  | -0.119 |  | * |
| Macrophage | CD68 | 0.037 |  | 0.391 |  | -0.008 |  | 0.869 |  | 0.084 |  | 0.059 |  | 0.044 |  | 0.332 |
|  | CD11b | 0.045 |  | 0.302 |  | 0.038 |  | 0.422 |  | -0.078 |  | 0.083 |  | -0.135 |  | * |
| M1 | NOS2 | 0.074 |  | 0.087 |  | 0.049 |  | 0.298 |  | 0.127 |  | * |  | 0.120 |  | * |
|  | CD86 | 0.013 |  | 0.758 |  | 0.003 |  | 0.946 |  | -0.012 |  | 0.796 |  | -0.067 |  | 0.144 |
|  | CD80 | -0.071 |  | 0.101 |  | -0.081 |  | 0.083 |  | -0.088 |  | 0.048 |  | -0.133 |  | * |
|  | ROS | -0.282 |  | *** |  | -0.260 |  | *** |  | 0.075 |  | 0.095 |  | 0.049 |  | 0.286 |
| M2 | ARG1 | -0.020 |  | 0.639 |  | -0.080 |  | 0.087 |  | 0.070 |  | 0.119 |  | 0.073 |  | 0.111 |
|  | CD163 | -0.064 |  | 0.143 |  | -0.090 |  | 0.053 |  | -0.016 |  | 0.719 |  | -0.067 |  | 0.144 |
|  | CD206 | 0.006 |  | 0.894 |  | -0.037 |  | 0.425 |  | -0.022 |  | 0.624 |  | -0.079 |  | 0.086 |
| TAM | HLA-G | 0.195 |  | *** |  | 0.185 |  | *** |  | 0.016 |  | 0.727 |  | -0.015 |  | 0.736 |
| Monocyte | CD14 | -0.161 |  | ** |  | -0.180 |  | ** |  | -0.090 |  | 0.043 |  | -0.163 |  | ** |
|  | CD16A | -0.002 |  | 0.955 |  | -0.010 |  | 0.829 |  | -0.059 |  | 0.191 |  | -0.105 |  | 0.022 |
|  | CD16B | 0.064 |  | 0.138 |  | 0.033 |  | 0.479 |  | 0.131 |  | * |  | 0.092 |  | 0.045 |
| NK | XCL1 | -0.027 |  | 0.528 |  | -0.027 |  | 0.568 |  | -0.095 |  | 0.034 |  | -0.091 |  | 0.046 |
|  | CD56 | -0.371 |  | *** |  | -0.355 |  | *** |  | 0.007 |  | 0.869 |  | 0.015 |  | 0.746 |
|  | KIR3DL1 | 0.141 |  | * |  | 0.103 |  | 0.026 |  | 0.053 |  | 0.240 |  | 0.035 |  | 0.452 |
|  | CD7 | -0.117 |  | * |  | -0.137 |  | * |  | 0.032 |  | 0.478 |  | -0.003 |  | 0.945 |
| Neutrophil | CD15 | -0.013 |  | 0.764 |  | -0.029 |  | 0.535 |  | -0.041 |  | 0.364 |  | -0.041 |  | 0.366 |
|  | CD66b | 0.022 |  | 0.619 |  | 0.003 |  | 0.951 |  | -0.006 |  | 0.896 |  | -0.008 |  | 0.861 |
| DC | CD1C | 0.098 |  | 0.024 |  | 0.086 |  | 0.066 |  | 0.038 |  | 0.396 |  | -0.013 |  | 0.781 |
|  | CD141 | -0.126 |  | * |  | -0.158 |  | ** |  | 0.155 |  | ** |  | 0.155 |  | ** |
|  | CLEC9A | 0.266 |  | *** |  | 0.256 |  | *** |  | 0.036 |  | 0.424 |  | -0.001 |  | 0.990 |
|  | XCR1 | 0.115 |  | * |  | 0.089 |  | 0.055 |  | 0.102 |  | 0.022 |  | 0.072 |  | 0.114 |
| Breg | CD1d | 0.010 |  | 0.811 |  | -0.030 |  | 0.519 |  | -0.075 |  | 0.094 |  | -0.121 |  | * |
|  | CD5 | -0.021 |  | 0.632 |  | -0.048 |  | 0.303 |  | -0.048 |  | 0.283 |  | -0.094 |  | 0.040 |

KIRC, kidney renal clear cell carcinoma; LUSC, lung squamous cell carcinoma. *P < 0.01; **P < 0.001; ***P < 0.0001.
