# Supplementary figures and images for: Prognostic and Immunological Value of Angiotensin-Converting Enzyme 2 in Pan-Cancer
Source: Front Mol Biosci. 2020 Sep 1;7:189. doi: 10.3389/fmolb.2020.00189 (PMC7490340; doi:10.3389/fmolb.2020.00189)

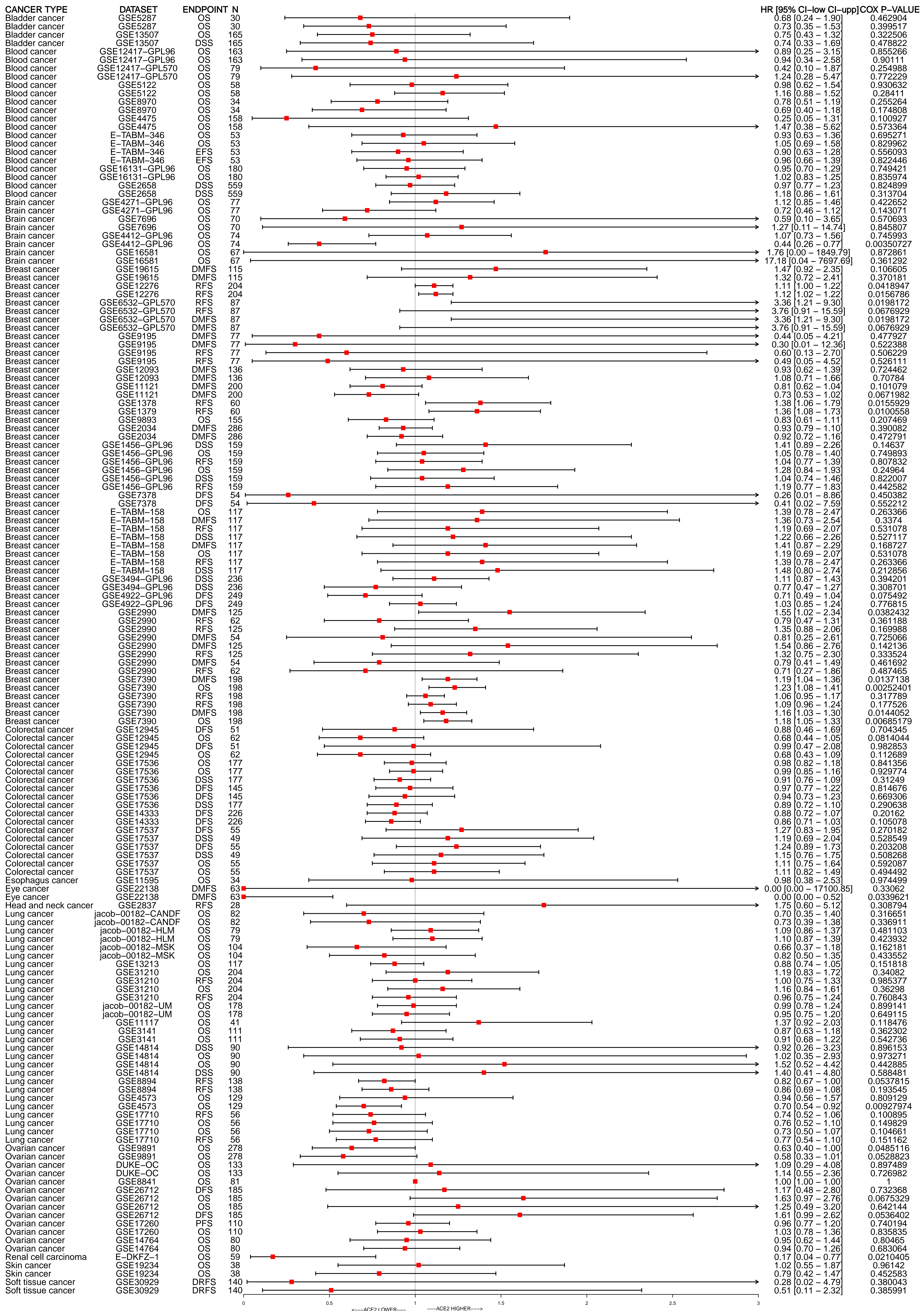

Supplement: Supplementary file 1 [file Data_Sheet_1.ZIP › Supplementary Figure 1.pdf]

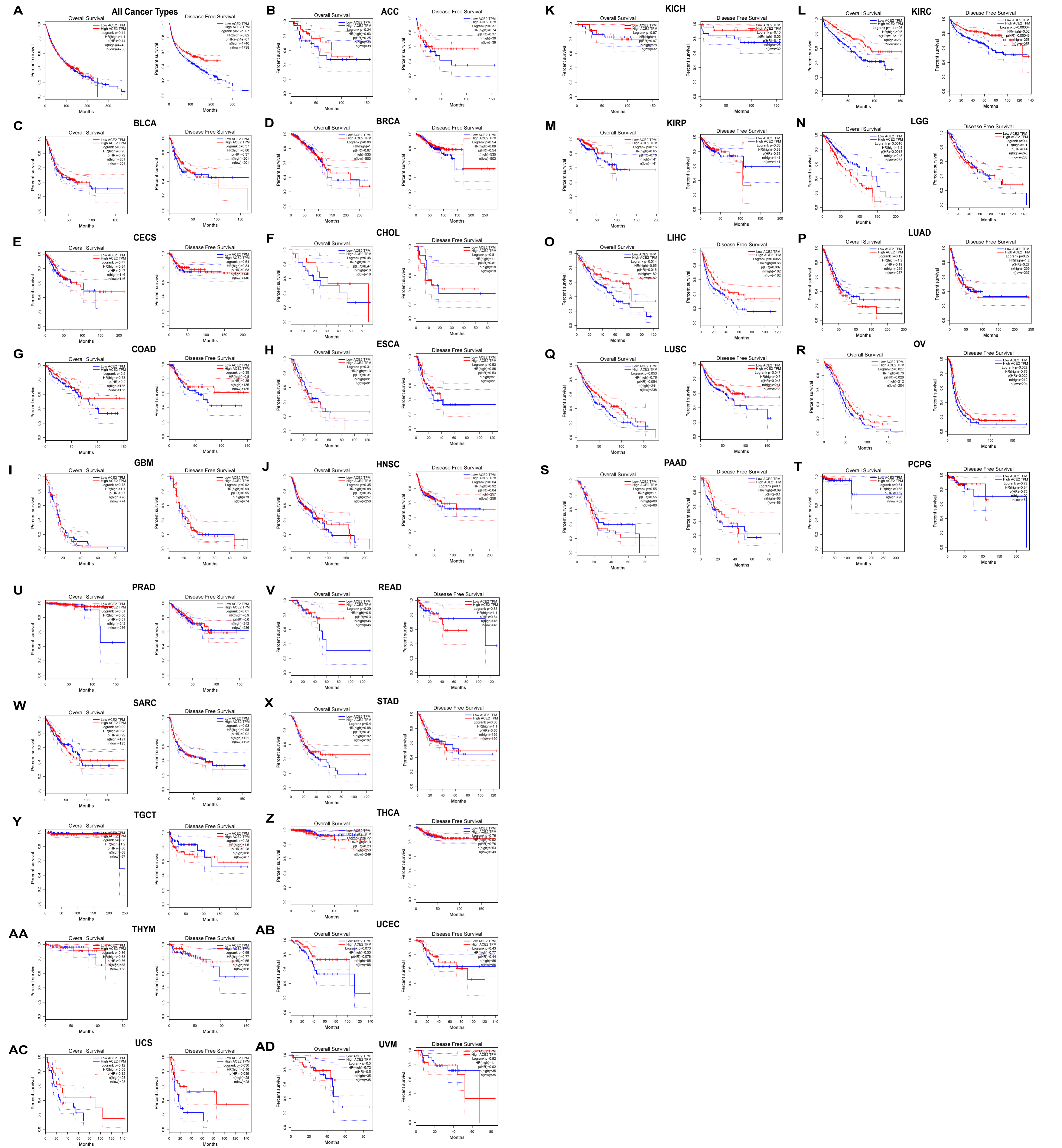

Supplement: Supplementary file 1 [file Data_Sheet_1.ZIP › Supplementary Figure 2.pdf]

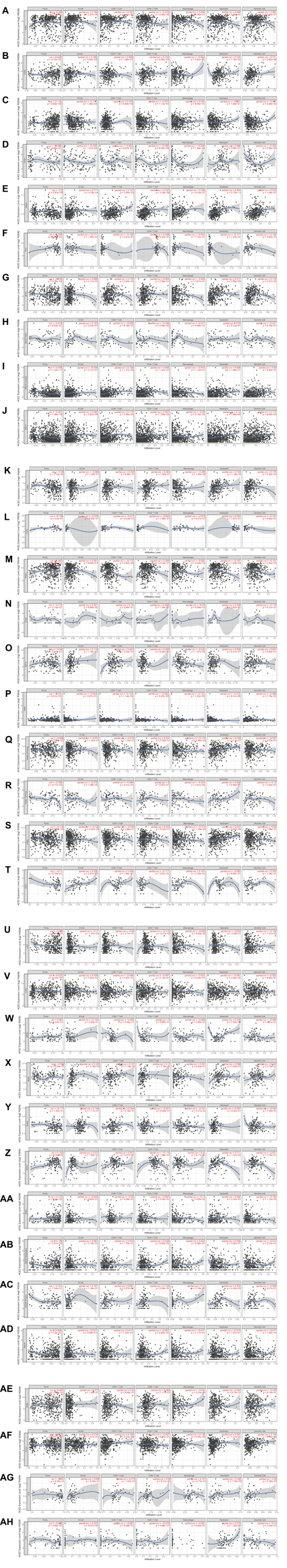

Supplement: Supplementary file 1 [file Data_Sheet_1.ZIP › Supplementary Figure 3.pdf]
